# The Effectiveness of the Anteroom (Vestibule) Area on Hospital Infection Control and Health Staff Safety: A Systematic Review

**DOI:** 10.3389/fpubh.2022.828845

**Published:** 2022-04-26

**Authors:** Elham Andalib, Masoumeh Faghani, Seyyed Mahdi Zia Ziabari, Mohammad Shenagari, Hamid Salehiniya, Mohammad Hossein Keivanlou, Zahra Rafat

**Affiliations:** ^1^Department of Design, Faculty of Fine Art, Music and Design, University of Bergen, Bergen, Norway; ^2^Clinical Research Development Unit of Poursina Hospital, Guilan University of Medical Sciences, Rasht, Iran; ^3^Department of Anatomy, School of Medicine, Guilan University of Medical Sciences, Rasht, Iran; ^4^Department of Emergency Medicine, School of Medicine, Guilan University of Medical Sciences, Rasht, Iran; ^5^Department of Medical Microbiology, School of Medicine, Guilan University of Medical Sciences, Rasht, Iran; ^6^Social Determinants of Health Research Center, Birjand University of Medical Sciences, Birjand, Iran; ^7^Student Research Committee, School of Medicine, Guilan University of Medical Sciences, Rasht, Iran

**Keywords:** anteroom, vestibule, airborne infection, nosocomial infection, SARS-CoV2, hospitalization, healthcare safety

## Abstract

The emergence of SARS-CoV2 in 2019 showed again that the world's healthcare system is not fully equipped and well-designed for preventing the transmission of nosocomial respiratory infections. One of the great tools for preventing the spread of infectious organisms in hospitals is the anteroom. Several articles have investigated the role of the anteroom in disease control but the lack of a comprehensive study in this field prompted us to provide more in-depth information to fill this gap. Also, this study aimed to assess the necessity to construct an anteroom area for hospital staff members at the entrance of each ward of the hospital, and specify the equipment and facilities which make the anteroom more efficient. Articles were identified through searches of Scopus, Web of Sciences, PubMed, and Embase for studies published in English until May 2020 reporting data on the effect of the anteroom (vestibule) area in controlling hospital infections. Data from eligible articles were extracted and presented according to PRISMA's evidence-based data evaluation search strategy. Also, details around the review aims and methods were registered with the PROSPERO. From the database, 209 articles were identified, of which 25 studies met the study criteria. Most studies demonstrated that an anteroom significantly enhances practical system efficiency. The results showed that the equipment such as ventilation system, high-efficiency particulate absorption filter, hand dispensers, alcohol-based disinfection, sink, mirror, transparent panel, UVC disinfection, and zone for PPE change, and parameters like temperature, door type, pressure, and size of the anteroom are factors that are effective on the safety of the hospital environment. Studies demonstrated that providing an anteroom for changing clothing and storing equipment may be useful in reducing the transmission of airborne infections in hospitals. Since the transmission route of SARS-CoV2 is common with other respiratory infectious agents, it can be concluded that a well-designed anteroom could potentially decrease the risk of SARS-CoV2 transmission during hospitalization as well.

## Introduction

The SARS-CoV2 has been at the forefront of all infectious agents that humans have ever encountered in terms of socioeconomic impacts. In most countries, the number of patients hospitalized with COVID-19 was more than the ICU bed capacity and the risk of hospital-acquired infections among healthcare staff is higher than ever. Hospitals play an essential role in responding to communicable disease epidemics and infectious disease control. In the case of COVID-19, the focus of the hospitals is on symptomatic patients and isolating them. Face masks, gloves, long-sleeved gowns, and goggles are known as personal protective equipment (PPE) in dealing with the spread of infectious agents (1–3). However, asymptomatic and presymptomatic individuals may serve as a reservoir in hospital-acquired infections including SARS-CoV2 (4, 5).

It was found that reinfections of some pathogens such as SARS-CoV2 among healthcare workers would more likely to be asymptomatic. So, asymptomatic healthcare workers can cause person-to-person transmission and should be considered as a source of infection in hospital wards to prevent potential outbreaks and the infection of different types of patients. Therefore, all necessary designs and facilities to minimize the risk of nosocomial infections should be considered especially in the respiratory isolation wards.

Hospital staff members work in the wards and are in direct contact with other staff members and asymptomatic patients and are more likely to be infected at work. On the other hand, infected hospital staff without any symptoms can infect their family members, other healthcare personnel, and visitors through person-to-person transmission, and also *via* indirect contact transmission involving contaminated objects or surfaces (6, 7). In addition, infectious microscopic organisms can transmit through the air in an indoor environment when the infected people produce respiratory droplets by breathing, talking, coughing, and sneezing (8–10).

It seems that in hospitals the focus of infection prevention programs is on preventing infection transmission through direct contact, and the indirect transmission of infections in hospitals has been neglected, except for isolation rooms (4).

One of the most important concerns of hospital staff members is the transmission of infectious organisms from the work environment to their homes and other wards (11). Factors such as reluctance to use leisure time due to the possibility of getting infected, fear of infecting others, lack of facilities to disinfect themselves and their equipment, and high load of airborne pathogens in the air of the hospital environment have caused excessive job burnout in medical staff members that endangers their physical and emotional health (12).

Scientific studies specified that well-designed physical settings can be an important tool in making hospitals safer and less risky and stressful for patients, their families, and hospital staff members (13, 14). The design of hospital physical environments should help to prevent the spread of infections and improve the safety, and quality of hospitals (12, 13). One of the great tools contributing to the prevention of infectious diseases is the anteroom (also known as vestibule) (15, 16). It is a hospital physical environment that is mostly used at the entrance of the isolation room and diminishes or prevents the spread of airborne infectious organisms and reduces the movement of contaminated air or other dangerous particles from the isolation room to the hallway (17). The anteroom is a negative or positive pressurized area containing cabinets for supplies, a sink for handwashing, and enough space for donning and doffing of PPE (18). The role of anteroom with negative pressure is different from that with positive pressure. Infectious patients' isolation rooms must be maintained at a negative pressure relative to the anteroom so that air flows into the room and not in the anteroom, theoretically preventing the escape of infectious aerosols from the isolation room. But the cancer patients' isolation room should be positively pressurized compared with the anteroom to prevent the movement of contaminated air to the isolation room (12–14, 17).

Several articles have investigated the role of the anteroom (vestibule) in disease control and prevention but until now there is no comprehensive study in this field. Considering the ongoing pandemic of Coronavirus, it was necessary to conduct a systematic review to provide a complete understanding and summarize the evidence associated with the effect of the anteroom (vestibule) area in decreasing hospital infections including SARS-CoV2 across and examine the necessity of reconsideration in the space division of hospitals to provide a healthier space for hospital staff members and patients. Also, this study aims to answer the following questions:

1- Is it necessary to construct an anteroom area for hospital staff members at the entrance of each ward of the hospital?

2- How is the effect of equipment such as ventilation system, high-efficiency particulate absorption (HEPA) filter, hand dispensers, alcohol-based disinfection, sink, mirror, transparent panel, UVC disinfection, and zone for PPE change, and parameters like temperature, door type, pressure, and size of the anteroom on the safety of the hospital environment?

3- What are the differences in the role of the anteroom with negative pressure from that with positive pressure?

## Methods

### Research Strategy

The results of this study were reported according to the PRISMA (Preferred Reporting Items for Systematic Reviews and Meta-Analyses) guidelines (19). Also, details around the review aims and methods were registered with the PROSPERO (registration number: CRD42021257048). We searched Scopus, Web of Science, PubMed, and Embase databases for articles regarding the effect of the anteroom (vestibule) area in controlling hospital infections. Searches were performed without date restriction until 7th March 2021. Articles written in English were searched. We also used references of included primary articles for search.

**Figure 1:**
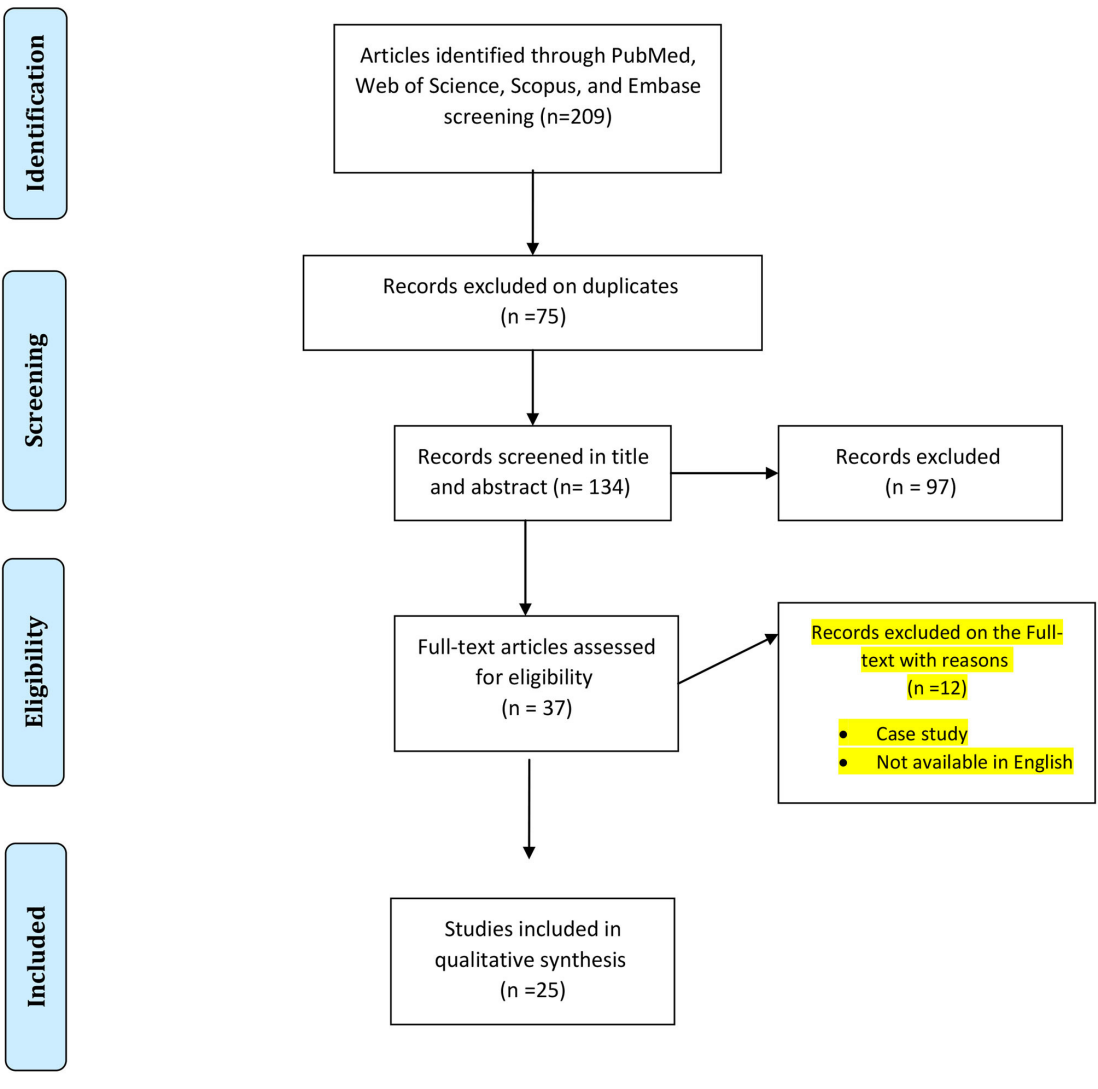
PRISMA flowchart showing the search and study selection strategy.

The search terms used were as follows: “anteroom OR vestibule,” AND “hospital,” AND “infection control.” Two independent researchers screened titles and abstracts of papers and any disagreements were resolved by a third reviewer. A PRISMA flowchart showing the study selection process is depicted in Figure 1.

### Inclusion Criteria

Studies were included in this review if they were peer-reviewed and written in the English language. Studies must have investigated the effect of the anteroom (vestibule) area in controlling hospital infections. Furthermore, studies must have clearly described the methodology and environments in which the study was performed.

### Data Extraction and Quality Assessment

All search results were managed with EndNote X7.1 (Clarivate Analytics, Philadelphia, PA, USA). Duplicates were deleted and the title and abstract of the remaining citations were reviewed to exclude irrelevant articles. For the remaining citations, full texts were downloaded and evaluated. Authors have independently assessed the risk of bias in the included studies, according to the criteria from the modified STROBE checklist, as a validated method for assessing the quality of observational and case-control studies (20). The instrument used a system to evaluate cross-sectional studies based on some criteria: title and abstract, background, objectives, study design, setting, participants, variables, data sources, and measurements, bias, study size, quantitative variables, statistical methods, participants' result, descriptive data result, outcome data and main results, other analyses, key results, limitations, interpretation, and generalizability (20).

For each domain, the following description was used for management of the risk of bias: “Yes,” “No,” and “Unclear.” We graded the quality of included studies and the risk of bias, using grading 1 for “yes” and 0 for “no and unclear,” and disagreement was resolved by discussion.

The following information/data were extracted from studies that met the inclusion criteria: name of the first author, year of publication, data collection period, continent, geographical location (country), study design, type of hospital infection (viral/fungal/bacterial), type of infectious organisms, interventions, study objectives, major findings, recommendation, and citation. When the reported data were insufficient or in the case of articles whose full text was not available, we contacted the corresponding by email to request additional information or full text. After three emails with a week's interval, these studies were excluded.

## Results

### Search Outcome

Systematically searching multiple data sources identified a total of 209 records. After the removal of duplicates, 134 papers remained and after screening by title and abstract 97 articles were excluded based on inclusion and exclusion criteria. Finally, 37 studies reporting data on the effect of the anteroom (vestibule) area in controlling hospital infections were assessed for eligibility and 25 study papers (21–44) were included in the systematic review after full-text scrutiny. The main characteristics of the included studies are summarized in Table 1.

**Table 1:** The detailed information presented in the 25 included studies.

**Row**	**First author**	**Year of Publication**	**Study period**	**Continent**	**Country**	**Type of study**	**Type of hospital infection**	**Type of microorganisms**	**Intervention**	**Objectives**	**Major findings**	**Recommendations**	**Study quality**
1	Ehsan S. Mousavi (21)	2020	NA	North America	USA	Original article	Viral	COVID-19	The effectiveness of the temporary plastic anteroom and the transportable air cleaner unit was accessed using an aerosolization machine with a surrogate oil-based substance.	To represent a novel temporary anteroom along with a transportable air cleaner unit to change an ordinary patient room into an isolation space.	- The temporary anteroom can prevent the migration of almost 98% of the aerosols into the adjacent corridor. - Inside the isolation room and close the patient's bed is the ideal place of a transportable air cleaner unit.	Inside the isolation room and close the patient's bed can be considered the best place for the air cleaner unit.	High
2	Jian Hang (22)	2015	NA	Asia	China	Original article	NA	NA	The probability of inter-cubicle airborne transmissions of pathogenic microorganisms through a shared anteroom was investigated using full-scale field measurements and CFD simulations. Tracer gas (SF6) was the main used equipment.	To estimate the probability of airborne transmission of pathogenic microorganisms in isolation cubicles that have a shared anteroom.	The presence of a shared anteroom used for multiple isolation cubicles can cause a remarkable inter-cubicle airborne transmission risk. The patients infected with different microorganisms should not be accommodated in isolation cubicles with a shared anteroom. The use of a swing door poses a risk in infection control.	For decreasing the inter-cubicle exposure hazards, reducing the period of the door opening, using a curtain at the doorway, and raising the air change rate were recommended.	High
3	Franceso M Fusco (23)	2012	February to November 2009	Europe	14 European countries (Denmark, Greece, Spain, Ireland, Austria, Slovenia, Bulgaria, Luxembourg, Finland, France, Malta, Italy, Norway, and Poland)	Original article	NA	NA	NA	To investigate capabilities for the management of highly infectious diseases in emergency and medical admission departments and present data about infrastructures and infection control procedures.	- Negative pressure for the isolation of patients, and use of HEPA filtration with the aim of exhausting air for isolations are among the main infection control procedures. - The presence of an anteroom increases the efficiency of the system.	- Implementing the transmission-based precautions, cough etiquette measures, and respiratory hygiene. - Early recognition, isolation, and management of patients. -Rapid information gathering about patient interactions and background. -Using personal protective equipment, disinfection equipment, and isolation. - Availability of well-equipped isolation rooms with anterooms, negative pressure, HEPA filtration, sealing doors, and windows, and easily decontaminate surfaces are fundamental.	Moderate
4	Christian Beauchêne (24)	2011	NA	Europe	France	Original article	NA	NA	- The treatment room and two anterooms including equipment, staff, and patient were considered as computational domain. - With the aim of comparison with the CFD observations, experiments with inert aerosol particles were performed in the prototype room. -Time-series measurement of airborne concentration in the anteroom and the treatment to validate airborne transportation.	To estimate the proper design of burn unit rooms via thermal convection flow.	- Thermal convention flows by the thermal difference between the treatment room and anteroom is an important factor. -Thermal convection can create contaminated zones close to the ceiling of the room. As a result, the contaminating transfer in adjacent rooms via thermal convection flows can occur which can bypass the protective overpressure in the patient room. -With a 2°C temperature difference between the anteroom and treatment room the risk of airborne contamination reduced. However, with higher temperatures, the transportation of air by opening and closing sliding doors increased.	- To compensate for the thermal convective flows the constructed burn unit can be equipped with supplemental air exhaust ducts over the doors. -Adopting the ventilation system design and room temperature to prevent microbial airborne transmission.	Moderate
5	Bård Venås (25)	2019	NA	Europe	Norway	Original article	NA	NA	Used a new system containing a fan that introduces filtered patient room air into the anteroom through a large displacement diffuser.	To analyze the air exchange by a hinged door movement and person passing the doorway and to assay a new technique to reduce airborne infection transmission in isolation rooms.	It was found that when a hinged door was opened, the new system stopped all transfer of air out from the patient room to the anteroom.	- The new system has the potential to be beneficial when considering inclusion in new standard AIIRs and to reduce the risk of contamination failure caused through person passing and door opening. -It is suggested to use procedural routines for passage or increasing the running time of airflow.	High
6	Ricardo Araujo (26)	2008	July 2004 to April 2005	Europe	Portugal	Original article	Fungal	*Penicillia, Aspergillus, Yeasts, Scedosporium, Alternaria, Cladosporium, Rhizopus, Mucor*	- Using an Andersen-type one-stage impactor the air of 18 rooms and wards equipped with different air filter systems, and access conditions were sampled.	To investigate the effect of different filters and access conditions in the transmission of airborne fungi.	The following factors were associated with lower fungal levels: HEPA filters, positive airflow, the existence of an anteroom, and the use of protective clothing.	The findings can be used in the hospital environment that high-efficiency filters cannot be installed.	High
7	EhsanS. Mousavi (27)	2016	NA	North America	USA	Original article	NA	NA	- To separating an isolation room from an anteroom. A doorway consisting of a single-hinged opaque door was used. -Ventilation was used to produce non-directional airflow. - Exhaust air was configured to produce directional airflow.	To Investigating the effects of door cycle speed and door motion on negative and neutral airflow between anteroom and isolation room.	- With increasing indoor cycle velocity, the air exchange increased. -The total volume of exchanged air was approximately equal to the swept volume of the door. - The directional airflow from the anteroom to the isolation room may disrupt and even reverse by door motion. - In the negative mode, the air release from the isolation room to the anteroom was 1-10 lower.	Other factors, such as door type (sliding vs. hinged) and swing direction, may be considered for future investigations regarding the optimum door design for hospital rooms.	Moderate
8	Petri Kalliomäki (28)	2016	NA	Europe, Asia	Finland and Singapore	Original article	NA	NA	- The airflow through the hinged door and sliding door with human passage was compared using a full-scale isolation room model. - In order to examine the different door operating parameters, the experiments were carried out in still air (i.e., without ventilation). -To illustrate airflow through the doorway smoke visualizations were performed.	To Show the differences between sliding and single hinged doors in airflow pattern.	- Compared to a hinged door, the sliding door induces a smaller air exchange through the doorway - A remarkable amount of air was transferred by the manikin from the isolation room.	Under the influence of realistic ventilation rates and pressure differences along with passage, the performance of the sliding and hinged doors can be compared.	Moderate
9	B. M. Andersen (29)	2006	NA	Europe	Norway	Original article	Bacterial	MRSA, TB, tuberculosis, and other airborne infections.	From surfaces of the anteroom, patient room, and bathroom microbial samples were obtained before and after irradiation with standard hospital environmental cleaning, UVC, and chloramine disinfection.	To determine the bactericidal effect of the ceiling- and wall-mounted UVC light on surfaces in isolation units, compared with standard hospital chemical disinfection.	The number of bacteria on surfaces directly or indirectly exposed to UVC was significantly reduced, as did 5% chloramine disinfection alone. Completely shadowed areas in the isolation unit but still required disinfection by chemicals.	- The effect of various UV doses on a wide range of pathogens can be studied. -The duration of irradiation required after which photoreactivation can no longer occur can be studied, too.	Moderate
10	David L. Johnson (30)	2009	NA	North America	USA	Original article	NA	NA	Into the interior of expedient-construction isolation modules equipped with a HEPA filter fluorescent 2-mm, aerosol particles were released.	The containment efficiency of expedient airborne infectious isolation units with and without anterooms in the absence and presence of care provider traffic was estimated.	- For all evaluated isolation configurations, the containment efficiency was excellent. (Exceeding 99.7%.). - With simulated traffic particle escape was statistically significantly more than without it - There was no statistically significant difference in particle escape with and without an anteroom	The containment under actual work use conditions and the effect of thermal conditions inside and outside the containment can be investigated.	High
11	Torsten Holmdahl (31)	2013	NA	Europe	Sweden	Original article	NA	NA	Description of experiences of new hospital environment planning.	To represent the experience of planning a new facility for infection control and to examine underlying theories associated with infection prevention and evidence-based design.	Single rooms with anterooms are necessary for infection control. Anterooms with high ventilation, positive pressure, and equipment such as hand disinfection help controlling infection transmission. The traffic flow and movement of patients with contagious diseases should be divided from other patients. Evidence-based design and technical solutions should be used to enhance flexibility during outbreaks.	A high proportion of single rooms in new hospitals is necessary. Hospital staff and other users attend all meetings of hospital projects. Advanced ventilation systems, equipped anterooms, hand hygiene, door access regulation system, and avoiding crowding are the most important factor to be considered in hospital environmental designing.	Moderate
12	Seo Yu Bin (32)	2015	8 June 2015–3 July 2015	Asia	South Korea	Original article	Viral	MERS	Using reverse transcription PCR and culture method environmental contamination from 4 patients in MERS-CoV units of 2 hospitals was investigated.	- To determine the potential role of environmental contamination by MERS-CoV in healthcare settings - To identify the period of viable virus shedding from MERS patients.	- MERS-CoV RNA was detected in samples from points and medical devices frequently touched by patients or healthcare workers in MERS patient rooms. -Viral RNA was detected up to 5 days from environmental surfaces following the last positive PCR from patients' respiratory samples. -Anteroom identified as a proper space for changing personal protective clothes and providing disinfection equipment -The anteroom that is disinfected frequently, plays a significant role in preventing virus transmission to corridor area.	The strict environmental surfaces, hygiene practices, and sufficient isolation period based on laboratory results rather than solely on clinical symptoms may be considered for future investigations. Developing a broad procedure for changing and removing PPE. Placing a mirror in the anteroom for assisting health staff in checking and controlling their PPE.	High
13	Victoria J. Fraser (33)	1993	NA	North America	USA	Original article	Bacterial	Tuberculosis	NA	- To specify the number and efficacy of hospital ventilation systems and investigate the mechanisms in place for evaluating the function of these systems in St. Louis hospitals.	−0.4 to 93% of each institution had isolation rooms. - Intensive care respiratory isolation rooms were presented in only 3 of 7 hospitals. - Regarding the percentage of designated isolation rooms there was a significant difference among hospitals.	Starting a routine program of testing isolation rooms and especially in the time that a patient is placed in respiratory isolation is recommended for all the institutions. - Close communications between hospital and plant engineering staff using original blueprints are recommended.	Moderate
14	G Ippolito (34)	2005	NA	Europe	Italy	Original article	Viral	Smallpox and VHF	NA	Demonstrated the response model of the Istituto Nazionale per le Malattie Infettive Lazzaro Spallanzani (INMI), Italy, and Rome for managing patients suspected of or infected by smallpox or VHF.	In the past two decades, INMI has efficiently controlled the spread of the HIV pandemic and also multi-drug resistant tuberculosis. Data from hospitals in other countries have shown that sporadic cases of VHF can be prevented by well-prepared systems.	A healthcare system with the ability to rapidly expand beyond normal services is recommended.	High
15	Jiyeon Park (35)	2020	NA	Asia	South Korea	Original article	Viral	MERS	To perform surgical procedures on MERS-related patients, 2 of 25 operating rooms in the main operating suite of the hospital were temporarily converted into negative-pressure operating rooms.	To share the infection control measures for surgical procedures on MERS-related patients.	The number of hospitals equipped with negative-pressure operating rooms is very limited and therefore, the probability to experience a MERS or other outbreak is very high. Using an isolation room with−4.7Pa and Anteroom with−1.2 Pa, lead to a clean area with 2,500 particles which are lower than the target cleanliness (<10,000).	- When managing MERS-related patients regardless of their PCR results, enhanced PPE (N95 masks, surgical gloves, eye shields, and surgical gowns) is recommended - Changing the positive pressure rooms to negative pressure rooms to prevent infection.	High
16	Yun-Chun Tung (36)	2011	NA	Asia	Taiwan	Original article	Viral	Measle, Influenza, and rhinovirus	- A grid system was used to simulate CFD. -Full outdoor air was supplied into both the anteroom and the isolation room. (The pressure difference between the two rooms was set at −8 Pa.)	Using CFD with the Wells-Riley equation the spatial distribution of infection risk of airborne transmission infections in hospital anterooms and isolation rooms was predicted.	- Compared to a 0.002 mm^3^ door gap, a 0.003 mm^3^ door gap decreased the infection risk between the isolation room and the anteroom. -The optimum pressure difference between the anteroom and the isolation room is −8 Pa and a 12-air change per hour through air supply is ideal. -Ceiling supply and wall exhaust should be considered.	- Expanding the room arrangement to cover arrangements of windows, doors, and furniture is better to be examined for better results.	High
17	Steven J. Emmerich (37)	2012	NA	North America	USA	Original article	NA	NA	Different control strategies and design issues associated with room pressurization and filtration were examined using multizone airflow and contaminant transport simulations.	Providing an overview of the tools and methods that can be used to demonstrate the current hospital design Practices.	- Using a differential-based ventilation flow on hospital building leakage better captures the relevant airflow. -Anterooms, especially those equipped with additional UVGI air purifier systems can be effective tools for decreasing contaminant transport.	Better estimates of flow differentials across the zonal boundaries are recommended. -The effect of weather conditions in reducing the pressure differentials should not be neglected. -Using various filtration systems can play a significant role in preventing airborne transmission.	High
18	Tracey A. Herlihey (38)	2016	October and December 2014.	North America	Canada	Original article	NA	NA	- The appropriateness, the potential for errors, and ease of use of various combinations of PPE were assessed using usability testing. - To analyze participant feedback a qualitative constructivist approach was used.	To determine issues during donning and doffing of PPE, and its procurement criteria and design for infectious diseases.	- It was found that environmental factors, such as anteroom layout have an important impact on safety. - The need to design PPE as a complete system rather than mixing and matching components were identified.	The usability of PPE, the design of the environment in which the PPE is to be used, and the design of protocols should be considered in future studies.	Moderate
19	Ehsan S. Mousavi (39)	2016	NA	North America	USA	Original article	NA	NA	- The transport of aerosol from a general patient room and nurse station to a nearby airborne infectious isolation room was assessed in an actual hospital. -The difference between negative and positive pressure in the anteroom and the way that it affects the prevention of infection transmission was analyzed. -A CFD model was developed to study the impact of positive pressure.	To investigate the risk of secondary infection for the patient in the isolation room and to appraise the role of anteroom when surrogate particles are released outside of the isolation room.	A positive pressure anteroom reduces the concentration of particles and disrupted the particles pathway. The risk of infection transmission in anterooms with positive pressure is lower than negative pressure anteroom.	A study on the effect of door motion and HCW movements is considered for future studies.	High
20	Anna Kokkonen (40)	2013	NA	Europe	Finland	Original article	NA	NA	By examining the contaminant removal efficiency, air change rate, and leakage of contaminants outside the isolation room the ventilation performance of AIIRs was investigated in 3 Finnish hospitals. The tracer gas technique was used for assessment.	To investigate the old and newly built AIIRs and anterooms regarding the performance of engineering controls in Finnish hospitals.	Despite high ventilation rates in the AIIR and anteroom infectious agents can escape from the AIIR during egress.	To improve the quality of AIIRs their performance should be tested regularly and the air distribution and removal efficiency of impurities in AIIRs and anterooms should be considered.	Moderate
21	Joon Kee Lee (17)	2020	NA	Asia	South Korea	Original article	Viral	COVID-19	With partitioning the isolation room an experimental anteroom was implemented. For reducing the risk of infection transmission negative air machine, mirror, sink, doffing space, and transparent panel were used.	Emphasize some crucial points and share experience in building temporary AIIRs for COVID-19 patients with severe signs and symptoms.	The anteroom has an important role in infection control in AIIRs and is a buffer zone between the patient's room and the corridor. The pressure difference of −8 Pa to −15 Pa was the optimum pressure gradient for anterooms. It helps health workers to control and remove their PPE. The disinfection of medical devices and sample bottles happens in the anterooms.	In centers admitting coronavirus-related patients more airborne-infection isolation rooms and anterooms are needed and wards and rooms must be carefully checked to ensure an ample supply of medical air and oxygen. During the crisis implementing anterooms in the isolation area by using partition would be a proper answer to infection control.	High
22	Noah J. Adams (41)	2011	NA	North America	USA	Original article	NA	NA	To simulate infectious droplet nuclei fluorescent microspheres were released into the AIIR. Airborne concentrations were measured inside the AIIR, in the anteroom, and at the corridor–anteroom door both with and without care provider movement through the AIIR. The experiment was conducted under different pressures of 2.5, 11, and 20 Pa. The other factor that was considered in the evaluation was the movement of health staff.	At differential pressures ranging from 2.5 to 20 Pa and under conditions of no provider traffic and simulated high provider traffic the containment efficiency in a properly designed anteroom-equipped hospital, AIIR was compared.	Consistent with previous studies, the containment effectiveness during provider traffic was decreased, Containment increased with increasingly negative pressure differential and decreased with increasing provider traffic	Additional studies of AIIR containment effectiveness under real-world patient care conditions, in the presence and absence of an anteroom, would provide the more detailed information needed to consider further refinements to AIIR design standards.	High
23	Tracey A. Herlihey (42)	2017	February and April 2015	North America	Canada	Original article	Viral	Ebola virus	A mixed-methods approach including human-factors usability testing and qualitative questionnaire responses was used. For this aim, a patient room and connecting anteroom were constructed.	The effectiveness of environmental design on doffing PPE in a simulated healthcare environment was investigated.	Outlining disposal bin locations, providing handrails to assist with doffing, securing disinfectant wipes and hand sanitizer; increasing prominence of color-coded zones, providing mirrors; and restricting the space to doff were deemed effective for healthcare workers.	A comprehensive study should be conducted on the impact of the environment on healthcare safety.	Moderate
24	Barbara Bannister (43)	2009	2003–2007	Europe	UK	Original article	Viral	Respiratory virus and VHF	Guidelines for high-level isolation unit was developed through gathering questionnaire information and expert's suggestion.	Gathering data about Nine categories for guidelines. (1) Operational and clinical management. (2) Work environment health and safety. (3) Diagnostic services. (4)Patient transport. (5) Ventilation and air system. (6) Clinical waste management. (7) Environment and equipment disinfection. (8) Design and construction for a unit. (9) Design and construction for isolation room	Innovative design can reduce the cost, risk of infection, enhance maintenance, and improve staff health safety. HLIUs contribute in patients and staff's safety and protects other wards and community by proper guidelines and protocols.	A collaborative approach and seeking expert's suggestionS are necessary for developing, maintaining, and achieving high-quality services.	High
25	Jan Styczynski (44)	2018	April to December 2012	Europe	NA	Original article	NA	NA	A questionnaire with five sections about air filtration, air changes, maintenance, the combination of isolation, and the protective environment was designed. From 543 EBMT centers that were registered, 238 centers from 37 countries filled the questionnaires. 177 centers from 36 countries provided reliable information for assessment.	Determining the quality of current centers for the protective environment, reporting, and reaching an agreement for recommendations.	Protective environments have an important role in maintaining the safety of patients and staff. Communication between health staff and engineering services is necessary for practical infection prevention and efficiency in the hospital system.	A routine activity checking of performance by hospital nurses or cleaning staff or physicians enhances the efficiency of the system. Knowledgeable staff about the details and maintenance conditions of systems lead to a better protective environment.	High

*NA, not available; USA, United States of America; CFD, computational fluid dynamic; MRSA, methicillin-resistant Staphylococcus aureus; VHF, viral hemorrhagic fever; UVGI, Ultraviolet germicidal irradiation; PPE, personal protective equipment, HEPA, high-efficiency particulate absorbing; PCR, polymerase chain reaction; AIIRs, airborne infection isolation; HCW, healthcare workers; UV, Ultraviolet; Pa, pascal, MERS-CoV, Middle East respiratory syndrome coronavirus; HLIUs, high-level isolation units*.

### Year of Publication and Study Location

**Figure 2:**
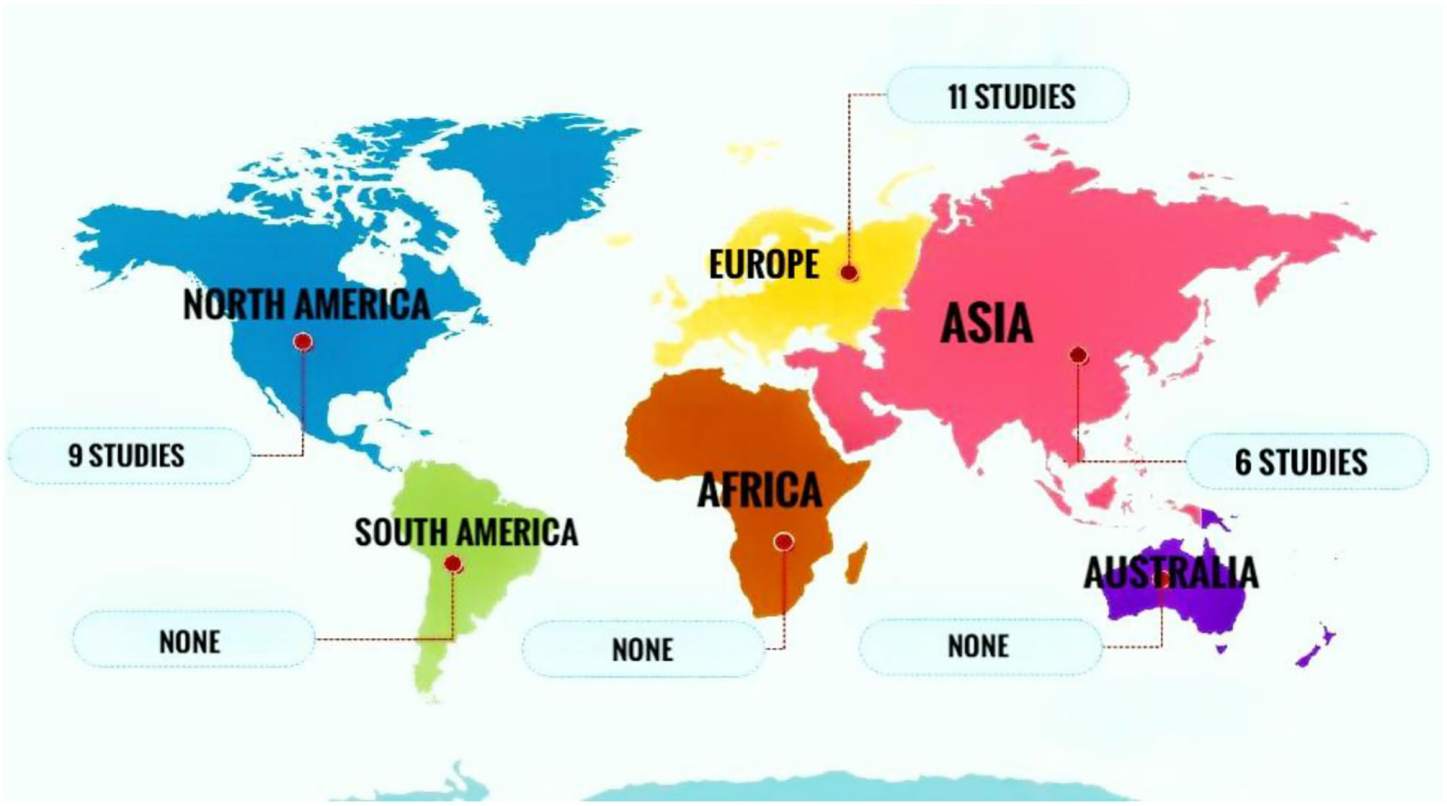
Several studies conducted in different continents reporting data on the effect of the anteroom (vestibule) area in controlling hospital infections.

All included papers were published between 1993 and 2020. One research reported data from 14 European countries (Denmark, Greece, Spain, Ireland, Austria, Slovenia, Bulgaria, Luxembourg, Finland, France, Malta, Italy, Norway, and Poland) as a single study (23). Results from a single study were showed data from 2 different countries (Finland and Singapore) (28). Also, data from one study was uncategorizable (44). Regarding the continent, the majority of the studies were conducted in Europe (*n* = 11) and North America (*n* = 9) (Figure 2), and regarding the country the highest numbers of reports were available for the USA (*n* = 7/25) and Nordic countries (*n* = 7/25).

### Type of Hospital Infection

Of the 11 studies reporting data for the effect of the anteroom (vestibule) area in controlling hospital infections caused by infectious organisms (17, 21, 26, 29, 32–36, 42, 43), 8 had separate data for viral infections (17, 21, 32, 34–36, 42, 43). Also, 2 studies reported data on bacterial infections (29, 33), and one study included fungal infections (26).

### Anteroom

Studies highlighted that an anteroom enhances practical system efficiency. It acts as a buffer zone and a layer between the isolation room and the corridor to maintain the pressure gradient and prevent pressure loss (17, 23, 32, 37). The anteroom is considered necessary for reducing the virus particle migration and air transmission from the isolation room to the corridor. It enhances environmental control and safety by reducing the risk of infection transferring (17, 32, 37). Frequently disinfection of the anteroom causes less virus transmission to corridors (32). The environmental design of the access point of hospital wards with spaces such as anteroom that are well-equipped and is able to exclude airflow, enhance infection transmission prevention and air load control (26).

### Factors Influencing Transmission of Infectious Disease Through the Anteroom

#### Pressure

Eleven studies highlighted the effects of pressure on preventing air transmission and reducing the risk of infection (17, 26, 27, 31, 35–37, 39, 41, 43, 44). Results of a study conducted by Jan Styczynski et al., demonstrated that among 177 centers from 36 countries, the numbers of existing centers which use positive and negative pressure for their anterooms pressure gradient are about the same number of 19–20% (44). Three studies mentioned that anterooms equipped with negative pressure prevent airborne transmission and high particle air load (26, 27, 35). In one study, it was reported that the air exchange during door opening and closing between an isolation room and anteroom with negative pressure of −2.5 Pa was reduced compared to the neutral situation (27). In another study, the positive pressure rooms environment was changed to negative pressure to prevent airborne transmission to near areas. The −1.2 Pa pressure was used for the anteroom environment (35).

Two studies emphasize that the positive pressure of an anteroom makes the system reliable and acts as an airlock to minimize the air and particle flow (31, 39). In one study, it was shown that the particles can transfer to the isolation room through the anteroom with negative pressure, while anterooms with positive pressure disrupt the particle flow way. The positive pressure acts as a barrier and reduces the concentration of particles (39). The other study mentioned that positive pressure was preferred to the negative pressure anteroom. The anteroom pressured set at 10 Pa. The pressure level of the room can be seen from the work area and nursing station (31).

Four studies focused on the pressure difference gradient between rooms (17, 36, 41, 43). In one study, a −8 Pa pressure difference between an anteroom and isolation room resulted in better function and effective infection prevention (36). In another study, it was mentioned that the pressure difference between the anteroom and the isolation room with the 20 Pa results in lower infection particle transmission than the difference pressure gradient of 1.5 Pa (41). In the other study, the pressure difference was −8 Pa up to −15 Pa based on the combination of the pressure difference between the isolation room and anteroom, and the difference between the anteroom and corridor (17). While a study reported a pressure difference of more than 15 Pa between anteroom and isolation room and between anteroom and other areas as a suitable pressure difference (43).

Four studies emphasized the practical and beneficial aspect of implementing the pressure device monitor for pressure checks (17, 31, 43, 44). A pressure device on the wall helps health staff to control the pressure difference easily and change air measurement (17, 44). A monitoring system for controlling the pressure difference between the anteroom and the patient room or anteroom and hallway to not fall under the determined gradient Pa would help to guarantee the maintenance of continuous positive pressure by alerting the staff (44). Checking tools such as visual pressure check gauges and alarms are set to control the performance of ventilation systems. Scheduled protocol plans are designed to have high prevention maintenance and keep the required standards (31).

#### Air Ventilation

Eleven studies considered that the air systems help to improve efficiency by removing particles (17, 21, 23, 26, 31, 33–36, 43, 44). The ventilation system of the isolation unit is separated from other wards and connected to a backup power source. The airflow and pressure gradient flow from the cleanest to the most contaminated area. The air does not circulate again and is exhausted in a site far from the building to reduce the risk of infection transmission for occupants and the community (43). An anteroom is ventilated by the incoming and outgoing air device (31). The location and type of air device have an impact on directing airflow and reducing the infection transmission (17). Air intake and air exhaust happen at two opposite sides of the room (44).

The rate of air exchange was examined by five studies. One study indicates that the rate of air exchanges between 5 and 10 times per hour is efficient, based on the type of patients. The normal situation required 5 times and 10 times per hour was used for patients with airborne transmission disease (31). Another study reported that an airflow exchange in an anteroom between 14 and 18 times per hour was effective (35). Two studies considered a minimum of 12-air exchange per hour to be mandatory for having an efficient ventilation system (43, 44). Room ventilation of 30 min was used to clean the area after a high-risk connection with the infected patient (35). The other study stated that the implemented air supply on the ceiling and wall exhaust that function with the 12-air rate change per hour helped in the extraction of contaminants. The increase in the number of air supply is not beneficial (36).

#### High-Efficiency Particulate Absorption (HEPA) Filters

Five studies emphasized that a HEPA filtration system enhances infection air load prevention (23, 26, 33, 34, 44). Anterooms that are equipped with HEPA filtration system at positive airflow showed a very low level of particles (26). HEPA filtration systems discharge the air, protect the environment, and individuals (23). One study mentioned that the desirable function system was achieved when the filters were checked, cleaned, and replaced frequently. Correcting the position and checking the function of dampers and controlling the exhaust ventilation is necessary (33). HEPA filters with an efficiency rate of 99.97% are able to remove particles ≤ 0.3 μm in diameter. Having a procedure for regular replacement and maintenance of filters by staff to control the efficiency of filters is suggested (44).

#### Size

Four studies emphasized the efficient and appropriate size of the anteroom to enhance its capability and usability (30, 31, 38, 42). The small size of the anteroom without ventilation and with basic equipment causes a pause during transit and increases the particles transmission (30). The anteroom physical environment and proper size of the anteroom provide the potential to have zones in the anteroom area and divide the zones into spaces to enhance infection control (38, 42). The standard size of the anteroom makes it possible to store PPE and supplies (31).

#### Temperature

Two studies pointed out that temperature plays a significant role in controlling airborne contamination. The 2°C temperature difference between the anteroom and isolation room significantly affected the air transfer, especially in the rooms which had sliding doors. When the difference between the two rooms' temperatures is more than 2°C, the risk of transporting contaminated sources to the next room is higher due to thermal convective flows (24, 28).

#### Equipment

Eleven studies provided information about the effects of anteroom's equipment on infection control (17, 23, 29, 31, 32, 34, 35, 38, 42–44). The proper environmental design and the placement of equipment significantly affect the contamination risk (38). Anterooms that are well-equipped act as a protective safe barrier for health workers and patients for infection control (34). Seven of eleven studies emphasized hand hygiene importance in the anteroom. Hand disinfection products such as a sink, hand dispensers, and alcohol-based disinfection are considered a protective process necessary for better healthcare workers' protection and prevention of infection transmission (17, 31, 32, 34, 38, 42, 44). In three studies, the placement of hand hygiene dispensers was considered an important factor. The hand dispensers should be implemented on the specific points of the wall which are secured, well-supported, and reachable (38, 42). Automatic dispensers are considered desirable and useful for healthcare workers. Putting several alcohol hand hygiene products is reachable and easy-access points enhance the infection control process (31). Using a hand-free sink for clinical use is recommended for healthcare staff safety (44).

Six studies emphasized the effect of the anteroom and its equipment for donning and doffing the PPE and its impact on infection transmission prevention. Healthcare workers use the anteroom as a place for applying or removing their PPE (23, 32, 35, 42). The donning and doffing area in the anteroom and implementing a border zone for separating this area from others reduce the infection risk and enhance the safety of the environment (35, 42). The suitable settlement of equipment provides a safe doffing situation such as the position of the removal bin that should be in arm's reach (38). Placing a hand railway in the area of donning and doffing, save the healthcare workers' balance and it is easier to disinfect. It also helps to manage and save space (17, 42). Using a mirror in the anteroom helps the medical staff to check and control their PPE material for patient care, anesthesia, medical devices, and sample bottles are placed or disinfected in the anteroom (17, 32, 34, 35). Having a transparent panel such as a window between the isolation room and anteroom enhances the safety of the environment since medical staff can communicate through the transparent panel and the doors are remained closed (17, 38). Windows in the wall or door help the staff to communicate or watch over the patient (44). One study stated that using UVC disinfection considerably decreases the bacterial population on surfaces that were exposed to UVC light directly or indirectly in a short exposure time. The usage of UVC for disinfection decreases the environmental bacterial infection and works as complementary chemical disinfection. The organisms that are not in the direct path of UVC light or in the shadowed area will not be destroyed and need chemical disinfection (42). The packages of equipment should be removed in a clean area and then transfer to the clinical area. Before disposal, solid waste should be disinfected. There are three methods that are suggested for liquid waste disposal, namely, solidification and autoclaving, disinfection with chlorine, and direct disposal to a dirty drain system (43).

#### Door

Fourteen studies stressed on the necessity and importance of door in the anteroom for infection control and the environmental safety (17, 21, 22, 24, 25, 27, 28, 31, 33, 34, 36, 38, 40, 44). The type of the door and the way that health workers use the door are factors that affect the pressure difference and performance of the anteroom (40). The air and particles exchange happens in the last second of the door closing cycle. With the increase in the door cycle speed, the airflow exchanges between the anteroom and isolation room increased significantly (27). The door of the anteroom should be closed to open the door of the next room. A 30-s pause is needed for stabilizing ventilation before the door opens (31). A self-closing door is recommended for maintaining a constant pressure gradient (44). Two studies emphasized the use of springs or manual systems that are applied to the door to control the unintended door opening and to reduce the door opening time (17, 33). In one study the door gaps between the isolation room and anteroom were shown as a factor that reduces the risk of infection. A 0.003 mm^3^ door gap reduces the risk of infection transmission compared to a 0.002 mm^3^ door gap (36).

Five studies focused on the importance of door types (21, 22, 24, 25, 28). Four studies indicated that swinging doors, also known as hinged doors weakens the function of the anterooms and poses a risk of infection. The possibility of infection transmission increased during the door rotation (21, 22, 25, 28). In one study it was mentioned that the use of a filter and fan system (airflow door barrier) near a hinged door area significantly removed the risk of contamination control failure by hinged door movement and reduced the infection risk, but not removed the person transmission. The sliding doors have less leakage compared to the hinged door (25). But both the sliding door and hinged door transfer significant airflow and pathogen from the isolation room to the anteroom and corridor. The volume of air exchange through sliding doors compared to hinged doors is notably lower. The duration of door-open had a relation to air transfer. When the total cycle increased, the amount of air volume exchange also significantly increased (28). In one study it was shown that when the sliding door is placed between the anteroom and the treatment room and the temperature difference is more than 2°C, thermal convection happens through the warm air gathered at the top of the door to the anteroom. So, significant contaminated air exchange between two rooms occurs. An air exhaust duct over the doors will be helpful to control thermal convective flows (24).

In general, according to the data presented in this article, we believe that there is sufficient evidence of an association between the presence of the anteroom (vestibule) area in each ward of the hospital and the decrease in transmission of airborne infectious diseases.

## Discussion

The COVID-19 pandemic and the lightning-fast spread of the extremely deadly virus SARS-CoV2 have impacted the lives of people all over the world. In previous studies, the airborne transmission of SARS-CoV2 has been confirmed (45, 46). The anteroom (vestibule) can be an essential tool to control airborne infections caused by viruses, bacteria, and fungi. Patient rooms, pharmacies, radiology suites, surgical suites, and cafeterias are all situations where an anteroom can help control the airflow between the sensitive patient-occupied area and other physical environments of the hospital. Despite its importance, until now there is no comprehensive study regarding the rule of the anteroom (vestibule) in disease control and prevention in hospitals. In this study, we reviewed all documents published in the English language on the topic across the world.

Our results showed that most of the studies included in this systematic review were conducted in the USA (*n* = 7/25) and Nordic countries (*n* = 7/25). This confirmed the fact that these countries are those in which much attention has been paid to the anteroom as an infection control area. Most USA hospitals use anterooms for hospitalized patients with suspected or confirmed airborne transmissible infections. In high-income countries like the USA and Nordic countries, 15% of hospital expenditure goes to the designation of hospital physical environments (e.g., anterooms) to prevent hospital-acquired infections (47). On the other hand, the results demonstrated that the application of anterooms in most countries, particularly low- and middle-income countries, is neglected. So, there is a need for a global platform to share knowledge in this field.

In this study, the predominant investigated infectious organisms in the majority of the imported studies fall into the category of viral infections (*n* = 8). Viruses, bacteria, and fungi are the etiologic agents of airborne diseases that are caused via transmission through the air. The main reason that in this systematic review viral infections contained most of the studies is the fact that in comparison with fungal and bacterial respiratory infections, respiratory viral infections are more contagious. In other words, in most cases, the mere demonstration of viable fungal or bacterial microorganisms in the air does not establish the occurrence of airborne transmission (48).

On the other hand, prior findings have emphasized the importance of an anteroom on health staff safety and efficiency of hospital systems. Anteroom's equipment has a significant effect on its performance and in reducing virus transmission. Equipment and parameters such as door type, air system, pressure, HEPA filter, temperature, hand dispensers, alcohol-based disinfection, sink, mirror, transparent panel, UVC disinfection, and zone for PPE change are factors that enhance the safety of the environment.

According to our investigation, pressure has a major effect on infection control (17, 26, 27, 31, 35–37, 39, 41). In the guidelines of different countries, various pressure gradients are suggested for the anteroom pressure between the range of negative and positive pressure. The USA, Nordic countries (Norway, Sweden, Finland, and Denmark), and Australia guidelines recommend an isolation room with negative pressure compared to the anteroom, and an anteroom with negative pressure compared with the corridor (47–49), while the UK recommends positive pressure for the anteroom compared to both the isolation room and the corridor (50). Some centers prefer the UK suggestion since an anteroom with positive pressure act as a barrier, blocks the air, and prevents air transmission (31, 51). As a reason, there are centers with different protocols for their anteroom. The centers differ in having positive or negative pressure and in the number of the pressure gradient (44). The various range of pressure gradients through all the centers indicate a need in revising the guidelines (51). The theory behind a negative pressure anteroom is that an anteroom with negative pressure acts as an empty space. So, when the door between an anteroom and corridor is opened, the air from the corridor with positive pressure transfers to the anteroom filling the space (26, 27, 35). By transmitting air from corridor to anteroom, pathogens existing in the hospital environment will transfer to the anteroom. If there is a leakage in an anteroom environment with negative pressure, air and pathogens from other areas would transmit to the anteroom. Then the polluted anteroom with higher pressure than the isolation room will be a danger to the patient who is vulnerable due to being infected with another virus and their weekend immune system (52). Positive pressure anteroom act like an airlock, preventing infection transmission from both corridor and isolation room entering the anteroom (31). Positive pressure anteroom reduces the fungal growth and protects the environment from infections ingress (53). So, the anteroom does not allow the infection from the isolation room to run to the corridor and vice versa. The same goes for the anteroom at the entrance of wards where staff could change their cloth and disinfect themselves after visiting patients. Positive pressure for this anteroom protects the healthcare staff and prevents infection transmission from the corridor to the safe area.

The range of pressure difference between isolation room and anteroom varied between 2.5 and 15 Pa (17, 36, 41), and was sometimes defined as 30 Pa (31). Several factors affect the maintenance of pressure difference such as door opening and closing (41, 54). The low-pressure difference puts a risk on maintaining the pressure condition and increases the risk of pathogens transmission from the isolation room space to the anteroom (55, 56). To ensure pressure difference maintenance and the safety of the anteroom environment, the higher-pressure difference will be more appropriate. It is recommended to set a monitor and alarm system for staff to check the pressure gradient of the environment regularly.

The ventilation system of the isolation unit is another factor that mostly affects the efficiency of the system (21, 23, 26, 31, 33–36, 40). The exhaust and intake air should be on the opposite sides of the room (44). The ventilation system of the isolation unit is separated from other wards and connected to a backup power source. The airflow and pressure gradient flow from the cleanest to the most contaminated area. The air does not circulate again and is exhausted in a site far from the building to reduce the risk of infection transmission for occupants and the community (43, 57).

The rate of air exchange per hour used for the anteroom environment varied between 5 and 18 times per hour (53, 58, 59). The rate of 5 and 6 is considered for a normal situation (60). The rate of more than 12 times per hour is mostly used when a patient with an infectious disease is hospitalized (61, 62). However, with viral infections such as COVID-19, patients may be asymptomatic or suspected to be infected. So, defining a normal situation would be doubtful. Setting the normal based on an air exchange more than 12 times per hour is required to have an efficient ventilation system and to be ensured patients' and healthcare staff's safety (43, 63). Room size is directly related to the amount of air conditioning per hour. As the volume of the room increases, the number of air changes also increases (64). The rate of air exchange for anterooms at the entrance of each ward should be regulated based on high human traffic.

High-efficiency particulate absorption (HEPA) filters are suggested to be used in the anteroom with the high efficiency at the exhaust air and are mandatory for places with the risk of circulation of exhaust air (23, 26, 33, 34, 43, 57, 65). The HEPA filters should be secured by prefilters and placed to have easy access for maintenance and checking (43, 66). Having a procedure for regular replacement and maintenance of filters by staff to control the efficiency of filters is suggested (44, 67). In the anteroom environment, HEPA filters are suggested for implementation on supply air (43). It seems that for the anteroom environment HEPA filters are used for both intake and exhaust air, and if the infrastructure is not accessible, portable HEPA filters would be a suitable solution (21). However, the portable filters might affect the circulation of airflow. Also, the regular changing and checking of filters have high importance in making a system reliable and efficient.

In addition, the temperature and size of the anteroom affect the factors mentioned above (68, 69). Increasing the temperature difference between the rooms, cause a convection current which increases the air transmission (24, 28, 70). It is recommended that the temperature difference between the anteroom and surrounding environments be minimized. The size of the anteroom plays an important role in infection prevention. The small anterooms do not provide a safe environment (71). The anteroom should be large enough to separate clean areas from contaminated spaces, such as spaces for used clothing, by zoning (38). Also, the anteroom should have places for storage of patient care equipment such as sampling glasses and staff PPE is required (42, 72). Therefore, it is suggested to set a standard for the anteroom space in the guidelines. So, people can have proper circulation in the designated zones and the possibility to do safety protection protocols.

A well-equipped anteroom assists the healthcare staff to enhance the efficiency and safety of the system (73). Most studies emphasize the practical usage of storage for supplies, PPE, mirrors, hand railways, hand-free sinks, several alcohol-based hand dispensers, transparent panels or windows, and zones for clean or contaminated areas (17, 23, 30–32, 34, 35, 38, 42). The anteroom is necessary for hospital staff to change and wear PPE, gloves, headwear, plastic footwear cover, and eye protection. Materials used for the patient should be disinfected with soap or bleach (74). A door or panel with a large sealed window is beneficial for healthcare workers for checking patients and communicating with other healthcare workers (59). Implementing UVC light for disinfection would be a suitable solution to disinfect anteroom areas in compliance with chemical disinfection materials as it reduces the air pollution while no one is present in the anteroom (75, 76). It is recommended that communication devices be placed between the two spaces so that people can talk through them and see the opposite side through the glass space to minimize the opening of the doors. For future research, researchers could investigate the UVC light implementation in the anteroom. Determining the number and distance of UVC lights based on the anteroom dimensions for maximum safety might be examined.

Another factor for maintaining the safety, pressure gradient, and preventing air transmission of anteroom is the door type (17, 18, 21, 24, 25, 27, 28, 31, 33, 34, 36, 38, 40). Research showed the use of sliding doors reduced the particle transmission between environments (77). Self-closing doors are recommended for applying in the anteroom (78). Also, when the two doors of the anteroom and isolation room are interlocked, it avoids air transmission and enhances safety and infection prevention (79). Using electronic identity cards for controlling access reduces the unnecessary door opening (59).

## Conclusion

In this study, we aimed to assess the necessity to construct an anteroom area for hospital staff members at the entrance of each ward of the hospital, and specify the equipment and facilities which make the anteroom more efficient. Based on our analyses, anterooms are effective in controlling infections and providing a suitable environment for staff's health and safety. For a reason, implementing spaces that perform and are equipped as an anteroom, and having a shower, at the entrance of each ward for health staff safety can significantly increase the safety of the healthcare staff at work. Furthermore, the results showed that the equipment such as ventilation system, HEPA filter, hand dispensers, alcohol-based disinfection, sink, mirror, transparent panel, UVC disinfection, and zone for PPE change, and parameters like temperature, door type, pressure, and size of the anteroom are factors that are effective on the safety of the hospital environment. Despite their importance, guidance on the construction of anterooms is less than clear. Also, the lack of education and communication about guidelines, infection control, and methods and equipment for safety such as PPE might cause problems and worries for healthcare staff. Setting an education about protocols and systems would be a helpful source to cope with crisis conditions. To the best of our knowledge, this study was the first comprehensive study to assess the necessity to construct an anteroom area for controlling airborne nosocomial infections during hospitalization and specify the equipment and facilities which make the anteroom more efficient. Since SARS-CoV2 share the common modes of transmission of most airborne viruses, namely, SARS-CoV, MERS-CoV, and influenza virus, it can be concluded that a well-designed anteroom could potentially decrease the risk of SARS-CoV2 transmission from person to person during hospitalization as during the current pandemic.

## Limitation

The necessity to construct an anteroom area in crowded places of hospitals, and specifying the equipment and facilities which make the anteroom more efficient in this situation was not described in this study that is suggested to be examined in future studies.

## Author Contributions

EA and ZR supervised the study and wrote the manuscript. MF, SZ, MS, and MK were involved in planning and supervising the findings of this work. HS contributed to the search strategy. All authors discussed the results, contributed to the final manuscript, and approved the submitted version.

## Conflict of Interest

The authors declare that the research was conducted in the absence of any commercial or financial relationships that could be construed as a potential conflict of interest.

## Publisher's Note

All claims expressed in this article are solely those of the authors and do not necessarily represent those of their affiliated organizations, or those of the publisher, the editors and the reviewers. Any product that may be evaluated in this article, or claim that may be made by its manufacturer, is not guaranteed or endorsed by the publisher.
